# Marine Antibiotics 2020

**DOI:** 10.3390/md19060351

**Published:** 2021-06-21

**Authors:** Yannick Fleury

**Affiliations:** Laboratoire de Biotechnologie et Chimie Marine, EA3884, Université de Bretagne Occidentale, Université Bretagne Sud, 29334 Quimper, France; fleury@univ-brest.fr

The range of environmental conditions in marine life is tremendous at different physico-chemical criteria (temperature, light, pressure and salinity). Since marine life has colonized all these ecosystems, the resulting marine biodiversity is complex to embrace in its entirety and has led to a prolific metabolic chemo-diversity. The global technological progresses (high-throughput DNA sequencing chemistry technologies coupled with powerful bioinformatic tools, and liquid chromatography coupled with high resolution mass spectrometry) will provide access to this gold-mine of bioactive compounds [1,2]. Therefore, marine life remains a reservoir and a source of inspiration for the development of therapeutically active molecules.

By now, the world emergency in term of public health is to re-prime the antibiotic pipeline in order to renew the anti-infectious therapeutic arsenal and to fight against antimicrobial resistance (AMR). Indeed, AMR is now recognised as one of the world’s biggest economic and security threats [3]. Despite considerable international efforts resulting from worldwide raising of awareness, international networks or incentives have been created to tackle AMR. Since July 2017, only 11 new antibiotics have been approved. Most of them belong to pre-existing classes and offer a limited clinical benefit. Moreover, the “2020 antibacterial agents in clinical and preclinical development” report from the WHO recently concludes that “Overall, the clinical pipeline and recently approved antibiotics are insufficient to tackle the challenge of increasing emergence and spread of antimicrobial resistance”.

By contrast, the preclinical pipeline is more dynamic and innovative. It includes 292 diverse antibacterial agents and a wider range of drug development projects. However, the success rate from preclinical stage to FDA approval is minimal. It has to be said that the antibiotic pipeline is almost dry. Antibiotic discovery has to be reinvigorated and marine biodiversity as an under-explored reservoir of bioactive compounds must play its full role in the race against time to tackle AMR.

This special issue, “Marine Antibiotics 2020”, gathers 5 publications, including a review, a communication and 3 research articles. The antimicrobial compounds described were characterized in the marine bacteria *Pseudoalteromonas* [4], the fungus *Penicillium citrinum* [5], the algae *Laurencia johnstonii* [6] and the cephalopod *Sepia officinalis* [7]. The authors have designed different strategies to identify the bioactive compounds (bio-guided fractionation, differential transcriptomic profiling). the antibiotic activity was highlighted against Gram-negative bacteria and Mycobacteria, two WHO critical priority pathogens. Finally, Nweze et al. draw up an inventory of the antimicrobial compounds isolated from marine from 2015 to 2019 on [8]. 

As guest editor, I hope that this special issue will illustrate the huge biotechnological potential of marine biodiversity. I would like to sincerely thank, of course, the authors, but also the reviewers who worked behind the scenes and without whom nothing would be possible, and finally, the editorial boards and the editorial office of *Marine Drugs* for their support and kind help.
